# Opioid-Free Anesthesia Versus Opioid-Based Anesthesia in Pediatric Dental Day-Case Surgery: A Retrospective Cohort Study of Safety and Efficacy

**DOI:** 10.7759/cureus.99793

**Published:** 2025-12-21

**Authors:** Ahmed A Khalaf, Ahmad Nabil, Hatem Ibrahim, Manjusha Bodhey, Zakia K Ahmed

**Affiliations:** 1 Department of Anesthesia, Dubai Hospital, Dubai Health, Dubai, ARE; 2 Department of Anesthesia and Intensive Care, Ain Shams University, Cairo, EGY

**Keywords:** analgesia efficacy, anesthesia safety, dental day-case surgery, opioid-free general anesthesia, pediatric anesthesia

## Abstract

Introduction

Opioid-free anesthesia (OFA) is increasingly utilized in pediatric surgeries to minimize opioid-related complications, such as postoperative nausea and vomiting (PONV) and delayed recovery. Its efficacy in pediatric dental day-case surgery remains underexplored. This study compares the safety and efficacy of OFA versus opioid-based anesthesia (OBA) in this setting.

Materials and methods

A retrospective cohort study reviewed medical records of 175 children (aged 3-10 years, ASA I/II) who underwent dental crowns/restorations between January 2024 and May 2025. Patients were grouped into OBA (n = 100) and OFA (n = 75) cohorts. Outcomes included postoperative pain scores, post-anesthesia care unit (PACU) duration, PONV incidence, rescue analgesic use, and unplanned admissions.

Results

Pain scores were 0.0 ± 0.0 in both groups at 0-2 hours and two to six hours post-surgery (OBA: 100/100, 100%; OFA: 75/75, 100%). PACU duration was significantly shorter in the OFA group (18.11 ± 6.88 minutes) compared to the OBA group (27.78 ± 11.03 minutes; p < 0.001). No patients experienced PONV (OBA: 0/100, 0%; OFA: 0/75, 0%), required rescue analgesics (OBA: 0/100, 0%; OFA: 0/75, 0%), or had unplanned admissions (OBA: 0/100, 0%; OFA: 0/75, 0%).

Conclusions

OFA provided equivalent pain control to OBA with faster recovery, supporting its use in outpatient pediatric dental surgery. Both approaches were safe, but OFA aligns with global opioid reduction efforts by minimizing opioid exposure.

## Introduction

Pediatric dental surgeries, typically day-case procedures under general anesthesia, have traditionally employed opioid-based anesthesia (OBA) for pain management. However, opioids are associated with adverse effects, including postoperative nausea and vomiting (PONV), respiratory depression, and prolonged recovery, which can impact patient safety and healthcare costs [1]. Opioid-free anesthesia (OFA), utilizing multimodal analgesia with agents like dexmedetomidine, acetaminophen, non-steroidal anti-inflammatory drugs (NSAIDs), and local anesthesia, has emerged as a promising alternative. While OFA has demonstrated efficacy in major pediatric surgeries, its use in minor procedures, such as dental surgery, remains less well studied [2]. The global opioid crisis has intensified the need to reduce opioid use, particularly in children, to mitigate risks of dependency and hyperalgesia [3]. This study evaluates the safety and efficacy of OFA compared to OBA in pediatric dental day-case surgery, focusing on pain control, recovery profiles, and complication rates.

## Materials and methods

Study design

**Figure 1:**
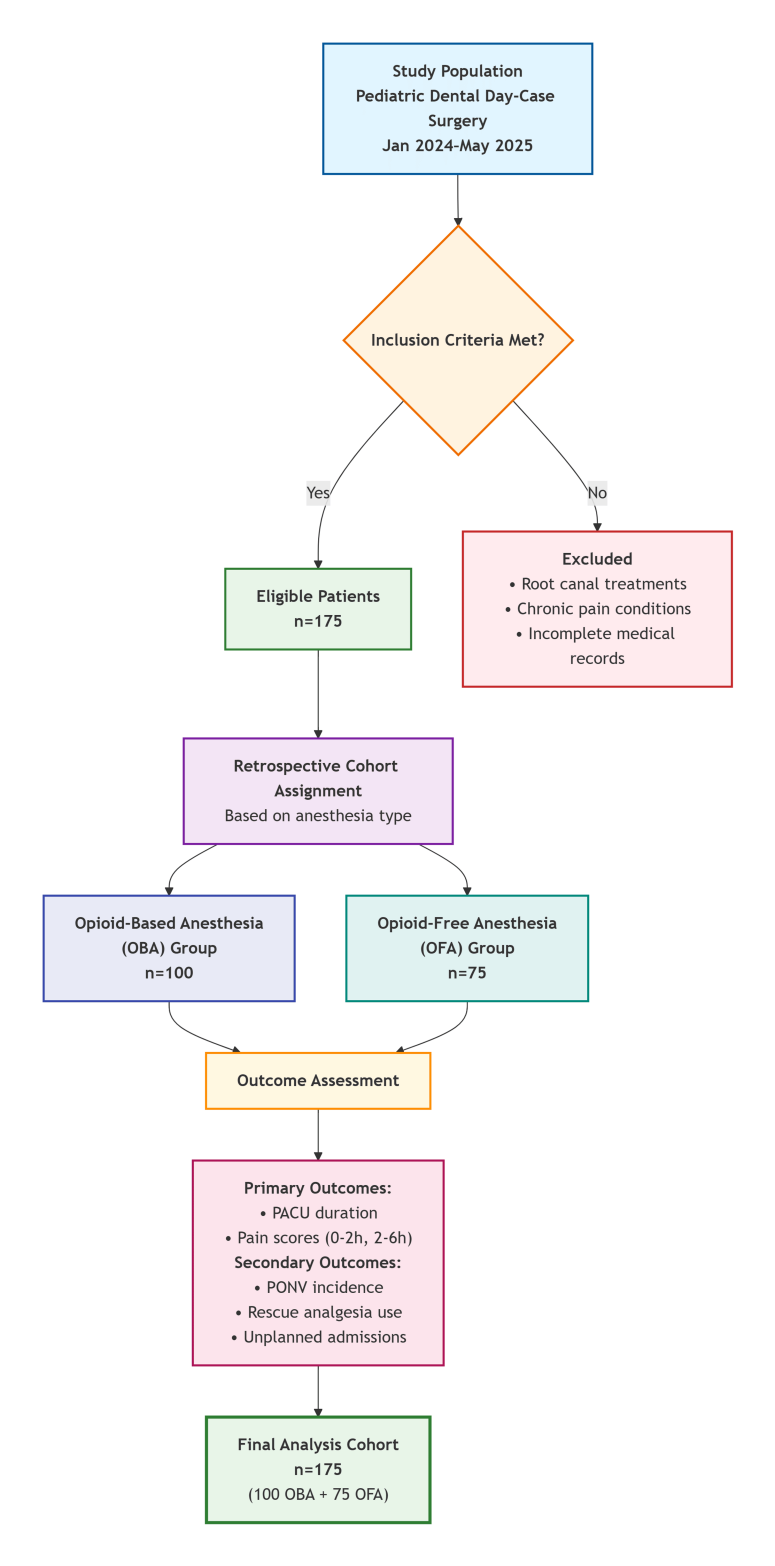
Flowchart showing patient selection and grouping process OFA: opioid-free anesthesia, OBA: opioid-based anesthesia, PACU: post-anesthesia care unit, PONV: postoperative nausea and vomiting

This was a retrospective cohort study conducted at Dubai Hospital in the United Arab Emirates between January 2024 and May 2025. The objective was to compare the safety and efficacy of OFA versus OBA in pediatric dental day-case surgery. The patient selection and grouping process is summarized in Figure 1.

Study population and sample size

A total of 175 children aged 3-10 years with American Society of Anesthesiologists (ASA) physical status I or II were included. Patients were divided into two groups: OBA (n = 100) and OFA (n = 75).

Sample size consideration

Given the retrospective nature of this study, a formal a priori sample size calculation was not performed. However, a post hoc power analysis was conducted based on the primary outcome, PACU duration. With the observed means (OBA: 27.78 minutes, OFA: 18.11 minutes) and standard deviations (OBA: 11.03, OFA: 6.88), the effect size (Cohen's d) was 1.04. For an independent t-test with α = 0.05 and power = 80%, the required sample size per group was 17. Our sample sizes of 100 (OBA) and 75 (OFA) provided ample power (>99%) to detect this clinically meaningful difference and to assess secondary outcomes.

The inclusion criteria were elective dental crowns or restorations lasting less than two hours. Exclusion criteria included root canal treatments, chronic pain conditions, or incomplete medical records.

Induction in both groups began with inhalational sevoflurane and oxygen via facemask, followed by intravenous access and administration of propofol (4 mg/kg IV) and ketamine (0.5 mg/kg IV). In the OBA group, fentanyl (2 mcg/kg IV) was added during induction, followed by tramadol (1 mg/kg IV) intraoperatively. In contrast, in the OFA group, a single bolus of dexmedetomidine (0.5 mcg/kg IV) was administered. After nasal intubation, anesthesia was maintained using a balanced technique with continuous propofol infusion (5 mg/kg/hour) combined with inhalational agents. Intraoperatively, both groups received intravenous paracetamol (10 mg/kg) and diclofenac (1 mg/kg IV infusion unless contraindicated), along with local anesthetic infiltration of lidocaine 2% with epinephrine 1:80,000, delivered in standard 1.8 mL dental cartridges, with a dosage of 4.4 mg/kg, with typical volumes ranging from 0.5 to 2 mL. At the operative site prior to surgery. Routine intravenous ondansetron was administered as antiemetic prophylaxis before extubation.

Study measures

Data collected included demographic characteristics (age, gender, and ASA status) and surgery duration. Postoperative outcomes included pain scores at 0-2 hours and 2-6 hours using the FLACC and Wong-Baker scales, post-anesthesia care unit (PACU) duration in minutes, incidence of PONV expressed as n (%), rescue analgesic use expressed as n (%), and unplanned admissions expressed as n (%).

Ethics statement

The study was approved by the Dubai Scientific Research Ethics Committee, Dubai Health Authority (approval number: DSREC-06/2025_02). It was conducted in accordance with the Declaration of Helsinki. Informed consent was waived due to the retrospective nature of the study.

Statistical analysis

Continuous variables were expressed as mean ± standard deviation and compared using the Mann-Whitney U test after assessing normality with the Shapiro-Wilk and Kolmogorov-Smirnov tests. Categorical variables were expressed as n (%) and compared using chi-square or Fisher's exact tests as appropriate. A p-value of less than 0.05 was considered statistically significant. Statistical analyses were performed using SPSS Statistics version 26 (IBM Corp. Released 2018. IBM SPSS Statistics for Windows, Version 26.0. Armonk, NY: IBM Corp.). Normality of continuous variables was assessed using the Shapiro-Wilk and Kolmogorov-Smirnov tests. Because key outcomes (PACU duration, age) were not normally distributed, nonparametric Mann-Whitney U tests were used for group comparisons.

## Results

The study included 175 patients (100 OBA, 75 OFA). Patient characteristics are summarized in Table 1. The OBA group had a mean age of 4.91 years (SD 1.46), 55.0% (n = 55) male, and 82.0% (n = 82) ASA I. The OFA group had a mean age of 5.59 years (SD 2.09), 57.3% (n = 43) male, and 73.3% (n = 55) ASA I. Surgery duration was comparable (OBA: 65.52 minutes, SD 13.94; OFA: 64.53 minutes, SD 15.84; p = 0.917). No significant differences were observed in gender (p = 0.758) or ASA status (p = 0.169).

**Table 1:** Patient characteristics by anesthesia type OBA: opioid-based anesthesia, OFA: opioid-free anesthesia, SD: standard deviation

Characteristic	OBA (n = 100)	OFA (n = 75)	p-value
Age (years, mean ± SD)	4.91 ± 1.46	5.59 ± 2.09	0.070
Gender (male, n, %)	55 (55.0%)	43 (57.3%)	0.758
ASA status (I, n, %)	82 (82.0%)	55 (73.3%)	0.169
Surgery duration (min, mean ± SD)	65.52 ± 13.94	64.53 ± 15.84	0.917

The distribution of continuous variables was assessed to guide the choice of statistical tests. As shown in Table 2, several key variables, including PACU duration in the OBA group and age in both groups, exhibited non-normal distributions (p < 0.05), justifying the use of nonparametric tests for group comparisons.

**Table 2:** Normality tests for continuous variables by anesthesia group Asterisk (*) indicates a lower bound of true significance. Given non-normality in key outcomes, non-parametric tests were appropriately used. OBA: opioid-based anesthesia, OFA: opioid-free anesthesia, PACU: post-anesthesia care unit

Variable	Group	Kolmogorov–Smirnov p	Shapiro–Wilk p	Distribution assumption
Surgery duration (min)	OBA	0.098	0.019	Non-normal
	OFA	0.200*	0.381	Normal
PACU duration (min)	OBA	<0.001	<0.001	Non-normal
	OFA	0.200*	0.184	Normal
Age (years)	OBA	<0.001	<0.001	Non-normal
	OFA	<0.001	<0.001	Non-normal

Pain scores were zero in both groups at 0-2 and 2-6 hours (p = 1.000). PACU duration was significantly shorter in the OFA group (18.11 minutes, SD 6.88) compared to the OBA group (27.78 minutes, SD 11.03; p < 0.001). No patients experienced PONV, required rescue analgesics, or had unplanned admissions (Table 3).

**Table 3:** Comparison of outcomes between OBA and OFA groups OBA: opioid-based anesthesia, OFA: opioid-free anesthesia, PACU: post-anesthesia care unit, PONV: postoperative nausea and vomiting, SD: standard deviation

Outcome	OBA (n = 100)	OFA (n = 75)	p-value
Pain score 0-2 h (mean ± SD)	0.0 ± 0.0	0.0 ± 0.0	1.000
Pain score 2-6 h (mean ± SD)	0.0 ± 0.0	0.0 ± 0.0	1.000
PACU duration (min, mean ± SD)	27.78 ± 11.03	18.11 ± 6.88	<0.001
PONV incidence (n, %)	0 (0%)	0 (0%)	1.000
Rescue analgesics (n, %)	0 (0%)	0 (0%)	1.000
Unplanned admissions (n, %)	0 (0%)	0 (0%)	1.000

**Figure 2:**
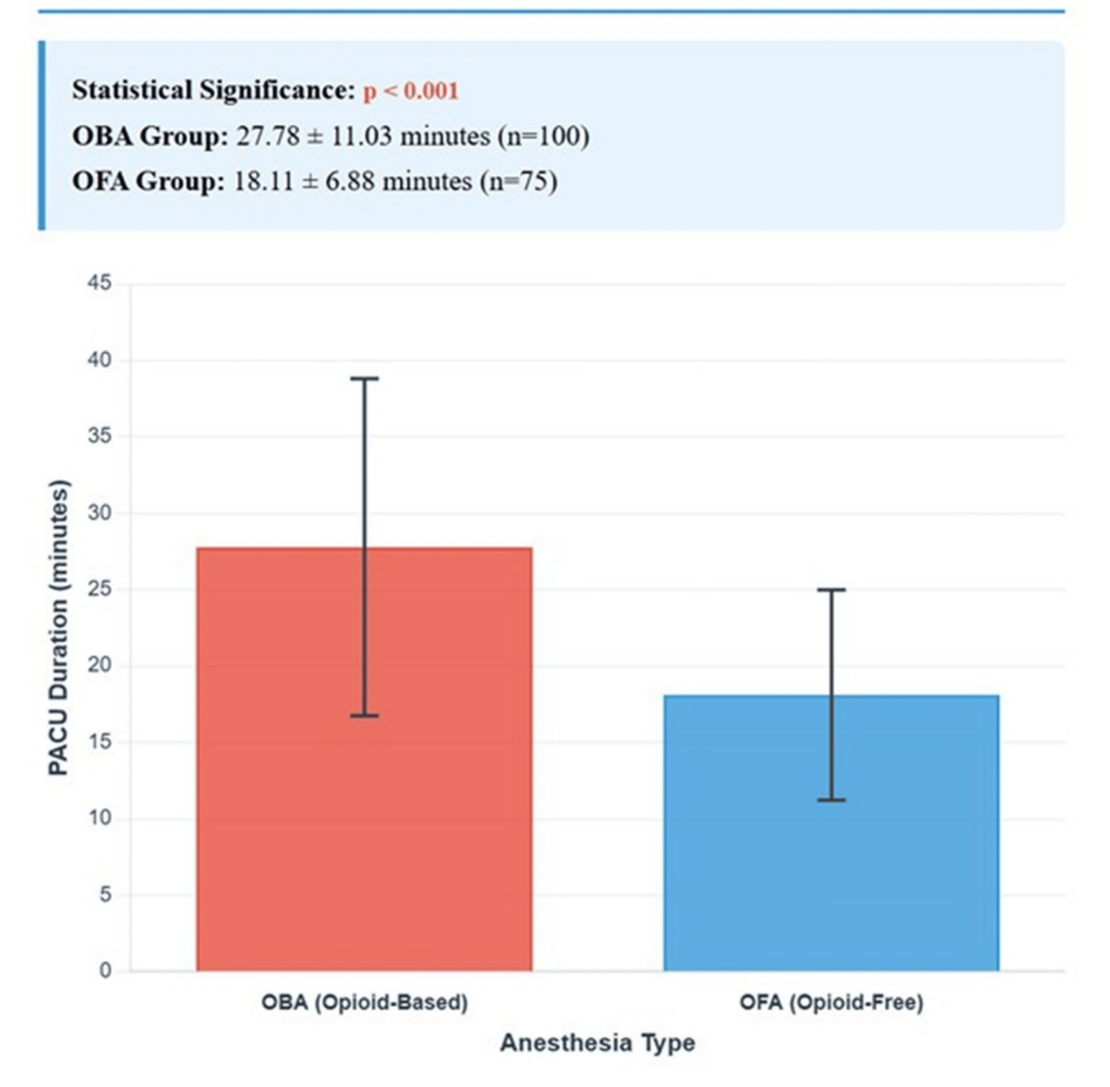
Bar graph of mean PACU duration by anesthesia type The OFA group demonstrated significantly shorter PACU duration compared to the OBA group (p < 0.001), while all other outcomes showed no statistically significant differences. OBA: opioid-based anesthesia, OFA: opioid-free anesthesia, PACU: post-anesthesia care unit

A bar graph comparing mean PACU duration (minutes) between OBA (27.78 minutes) and OFA (18.11 minutes). The x-axis represents anesthesia type (OBA, OFA), and the y-axis represents PACU duration (minutes). Error bars indicate standard deviations (±11.03 for OBA, ±6.88 for OFA). The graph highlights the statistically significant difference (p < 0.001), as shown in Figure 2.

**Figure 3:**
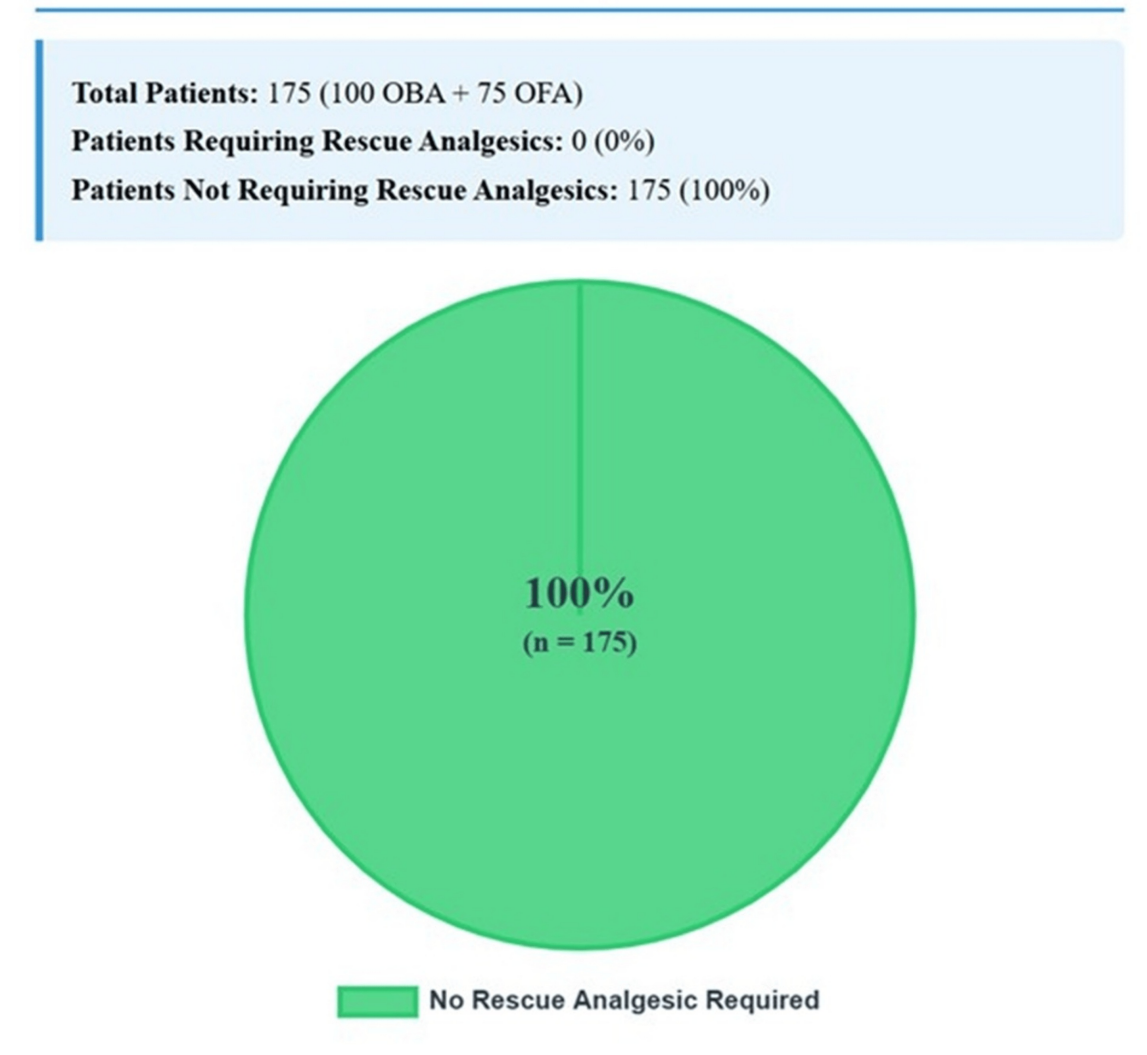
Pie chart of rescue analgesic use OBA: opioid-based anesthesia, OFA: opioid-free anesthesia, n: number of patients

Figure 3 shows a pie chart showing that no patients in either group required rescue analgesics (100% "no rescue analgesic" for both OBA and OFA). The chart uses a single segment labeled "no rescue analgesic" (100%).

## Discussion

This study demonstrates that OFA provides equivalent pain control to OBA in pediatric dental day-case surgery, with significantly shorter PACU durations. The absence of PONV, rescue analgesic use, and unplanned admissions in both groups underscores the safety of both approaches in this low-complexity setting. However, the faster recovery with OFA, as visualized in Figure 1, supports its adoption in outpatient pediatric anesthesia, aligning with global efforts to reduce opioid use amid the opioid crisis.

The zero pain scores at 0-2 and 2-6 hours in both groups indicate that OFA’s multimodal analgesia, incorporating dexmedetomidine, acetaminophen, NSAIDs, and local anesthesia, is as effective as opioids for minor dental procedures. This finding corroborates Eizaga et al. [4], who reported that dexmedetomidine-based analgesia effectively controls pain in pediatric surgery with minimal opioid use. The absence of rescue analgesic use in both groups, as shown in Figure 2, further supports OFA’s efficacy. However, zero incidence may reflect the low pain burden associated with crowns and restorations.

The significantly shorter PACU duration in the OFA group (18.11 vs. 27.78 minutes, p < 0.001) aligns with prior studies highlighting OFA’s role in enhancing recovery. Ma et al. [5] reported reduced recovery times in adult outpatient surgeries with OFA, attributing this to the avoidance of opioid-induced sedation and respiratory depression. Feenstra et al. [6] conducted a meta-analysis showing that OFA decreased PACU stays across surgical specialties, consistent with our findings. The sedative-sparing effects of dexmedetomidine and the absence of opioid-related side effects likely contributed to faster recovery in our OFA group, as noted by Beloeil et al. [7]. This has practical implications for day-case settings, where rapid recovery improves patient turnover and reduces costs.

The 0% PONV incidence in both groups was unexpected, given that Kovac [8] reported higher PONV rates with opioids in pediatric anesthesia. This may be attributed to the short duration and low complexity of dental procedures, which minimize triggers of nausea, and to the routine use of antiemetic prophylaxis (e.g., ondansetron) in both groups. Ames and Machovec [9] found that prophylactic antiemetics significantly reduce PONV in outpatient pediatric surgeries, supporting this interpretation. Future studies should explore whether OFA reduces PONV risk in settings with less aggressive antiemetic use.

The absence of unplanned admissions reflects the safety of both anesthesia techniques in this cohort. Franz et al. [10] reported low complication rates with OFA in pediatric ambulatory procedures, which aligns with our findings. However, the zero-admission rate may indicate a ceiling effect in minor surgeries, where complications are rare.

This study’s findings have significant implications for pediatric anesthesia practice. By demonstrating OFA’s efficacy and safety, it supports the shift toward opioid-sparing techniques advocated by Kharasch et al. [11], who emphasize minimizing opioid exposure in children to reduce risks of long-term dependence and hyperalgesia. OFA’s adaptability, as highlighted by Phillips et al. [12], allows for tailored protocols that can be standardized across institutions. Additionally, faster recovery with OFA may improve patient and parental satisfaction, though this was not assessed due to data limitations.

Clinical implications and recommendations

This study demonstrates that OFA provides equivalent pain control to OBA in pediatric dental day-case surgery, with the added benefit of significantly shorter recovery times. The absence of PONV, rescue analgesic use, and unplanned admissions in both groups underscores the safety of both approaches in this low-complexity setting. However, the faster recovery with OFA, as evidenced by a nearly 10-minute reduction in PACU duration, supports its adoption in outpatient pediatric anesthesia. The following clinical implications are significant:

(1) Enhanced operational efficiency: Shorter PACU stays can improve patient turnover in busy day-surgery units, potentially increasing capacity and reducing wait times. (2) Opioid-sparing practice: OFA aligns with global efforts to reduce perioperative opioid exposure, minimizing risks of opioid-related adverse effects, dependency, and hyperalgesia in children. (3) Safety in low-complexity surgery: Both techniques proved safe for pediatric dental procedures, providing clinicians with viable alternatives. (4) Standardization potential: The consistent zero pain scores suggest that OFA protocols can be standardized for similar outpatient pediatric surgeries.

We recommend OFA as a first-line anesthetic strategy for pediatric dental day-case surgeries, particularly in institutions pursuing opioid-reduction initiatives. Future implementation should include staff training on OFA protocols and monitoring of long-term outcomes, including patient satisfaction and cost-effectiveness.

Limitations

Several limitations must be acknowledged. The retrospective design introduces potential selection bias, as anesthesia type was not randomized. The single-center setting at Dubai Hospital may limit generalizability to other populations or healthcare systems. The zero pain scores, PONV rates, and rescue analgesic use suggest a ceiling effect in minor dental surgeries, potentially obscuring differences between OFA and OBA. The lack of data on parental satisfaction, as proposed in the study protocol, limits insights into caregiver experience. Specific OFA protocol components (e.g., dexmedetomidine doses) were not detailed in the records, hindering standardization. The short follow-up period (2-6 hours) may not capture delayed complications, such as late-onset pain or nausea. Finally, the sample size (n = 175) may be underpowered to detect rare events, such as admissions or PONV.

Prospective, multicenter studies with randomized designs are needed to confirm these findings and address selection bias. Standardized OFA protocols, as suggested by Franz et al. [13], could enhance reproducibility. Assessing long-term outcomes, such as pain control beyond six hours and neurodevelopmental effects of anesthesia, would provide a comprehensive evaluation. Including parental satisfaction scores and cost-effectiveness analyses could further elucidate the benefits of OFA. Larger sample sizes are needed to detect differences in rare outcomes, such as PONV or admissions.

## Conclusions

OFA provided safe and effective analgesia comparable to OBA in pediatric dental day-case surgery, with the added benefit of significantly shorter PACU recovery times. The absence of PONV, rescue analgesic use, and unplanned admissions in both groups underscores the safety of both approaches. At the same time, OFA’s opioid-sparing profile aligns with global efforts to reduce opioid exposure in children. These findings support OFA as a practical alternative to OBA in outpatient pediatric anesthesia, warranting further prospective, multicenter studies to validate long-term outcomes and broaden its application.
